# Mahaim Fibre Tachycardia: Recognition and Management

**Published:** 2003-04-01

**Authors:** Eduardo Back Sternick

**Affiliations:** Biocor Instituto and Hospital Vera Cruz, Belo Horizonte, Brazil

## Introduction

 Dr. Gallagher et al [1] wrote 22 years ago that "the role of Mahaim fibers in the genesis of cardiac arrhythmias in man has been controversial since they were first described " in the late 30's by Dr. Ivan Mahaim [2]. The very early reports were strictly anatomical studies [2-6]. This histopathologic quest did not end yet. Mahaim fibers were supposed to be accessory connections taking off from the His bundle and fascicles (FV-fasciculoventricular) to the right ventricle or from the atrioventricular node (NV-nodoventricular fibers) to the right ventricle. Anderson et al [7] proposed 2 varieties of NV fibers, one that arises from the transitional zone and the other which inserted from the deep, compact nodal portion of the AV junction. In his pioneering work HJJ Wellens paved the road for clinical electrophysiological investigation. He was the first to study a patient with accessory pathway with decremental properties and long conduction times assuming its relationship with the fibers described long ago by "Mahaim", as reported in his doctoral thesis [8] in 1971. The term nodofascicular (NF) was applied when the retrograde His bundle potential preceded the ventricular deflection, while nodoventricular pathway would be appropriate when the retrograde His bundle deflection followed the ventricular potential. It took some years to electrophysiologists realize the conceptual mismatch among the "Mahaim" physiology and structure described by Mahaim et al. An important observation was done in 1978 by Becker et al [5] who found an accessory node associated with a bundle of specialized fibers measuring 1 cm and coursing through the right ventricle, mimicking a second AV conduction system located on the lateral tricuspid annulus. However, that did not change the mainstream concept of NV fibers. During the early 80's many centers started to refer patients with drug refractory tachycardias to surgical treatment. According to the current concepts at that time targeting the A-V node would be the logic strategy for curative treatment of patients with NV/NF fibers. Some courageous electrophysiologists used a new technique consisting of high-energy catheter ablation of the A-V node to treat a patient with "Mahaim" fiber, which yielded complete AV block and persistent preexcitation [9]. The turning point came in 1988 at the University Hospital of Western Ontario, Canada, when Klein, Guiraudon et al [10] had decided to extensively freeze the A-V node and upper His bundle region of a 29 year old man and they soon realized that preexcitation did not go away. It became clear for them that his accessory pathway was not linked whatsoever to the A-V node. The next patient was luckier, and had kept intact his A-V node, while his "Mahaim fibers" were successfully severed after ice mapping produced a consistent zone of reversible block in the accessory pathway at the right lateral aspect of the tricuspid annulus. Klein's manuscript was received on August 24, 1987, and published the next year on JACC. Two months later (October 20, 1987) Circulation received a manuscript from Tchou P et al [11] entitled "Atriofascicular connection or a nodoventricular fiber? Electrophysiologic elucidation of the pathway and associated reentrant circuit". From a single case report we were taught how simple it is to make sure that such pathways arise from the atrium. In recent years catheter ablation techniques have shed more light on the subject. Discrete "Mahaim" potentials that are considered surrogates of pseudo-Mahaim tissue depolarization, are used as an effective target for ablation [12,13]. A number of pharmacologic [14] and histologic data [5,6,15,16], electrophysiologic maneuvers and observations during radiofrequency catheter ablation like heat induced "Mahaim" automaticity [19,20] are regarded as evidences of either an ectopic A-V node or remnants of the specialized A-V ring tissue. The NV/NF fibers are now considered a rare item but there are some convincing reports [21] of narrow and regular QRS tachycardias with ventriculoatrial dissociation. The last variety which is known as fasciculoventricular pathway [22] seems to play no role in clinical tachycardias but as long as it is very often associated with bypass tracts they should be correctly recognized and not targeted for ablation, avoiding unnecessary damage to the A-V node-His bundle conduction system.

## Recognition

### Electrocardiographic Features

**Figure 1:**
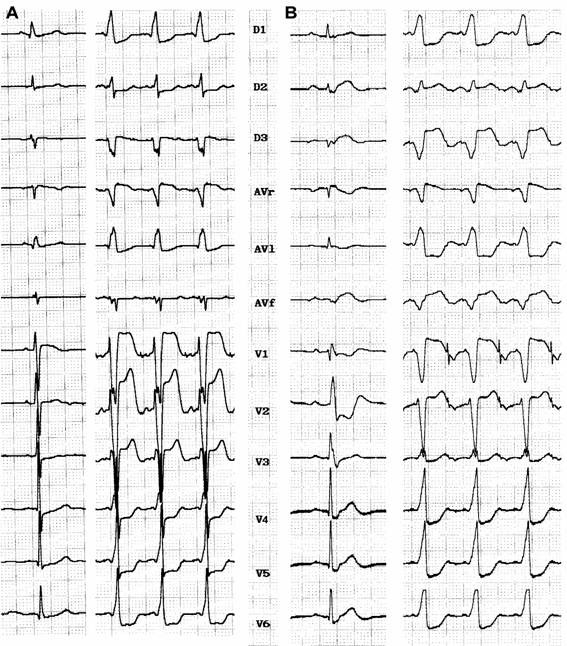
**A**: Atriofascicular pathway: baseline ECG discloses minimal preexcitation with a 0.12 s PR interval. During atrial pacing LBBB with âQRS= -20°, QRS 130 msec wide. **B**: Atrioventricular pathway in Ebstein's disease: baseline ECG without preexcitation and disclosing RBBB with normal PR interval. During atrial pacing manifest preexcitation with LBBB (âQRS= -30°), QRS 160 msec wide with a slurred initial r wave in V1

Baseline electrocardiogram of patients with atriofascicular or atrioventricular pathways (pseudo-Mahaim) (Figure 1) are characterized by minimal or no preexcitation. Sometimes the only clue is absence of septal Q wave in leads V5 or V6 [23]. Some patients show a typical LBBB with normal PR interval. A preexcited ECG is more likely to occur in an atrioventricular decremental pathway [24]. Precordial transition (R/S >1) usually occurs at V4 or V5 (sometimes V6). Latent preexcitation has recently been reported, in patients with spontaneous LBBB-like antidromic tachycardia, without preexcitation at rest and during atrial pacing [25]. A high degree of day-to-day variability as far as preexcitation is concern, the "concertina" effect is observed in many patients. Anterograde conduction over atriofascicular fibers yields a typical LBBB pattern with variable axis, superior frontal plane axis being the most commom one (ranging from -25° to -60°), but it is of no help in differentiating it from the atrioventricular pathways. QRS complex is usually larger with anterograde conduction over an atrioventricular pathway, with a slurred QRS onset [26] due to distal muscular insertion, which can be better appreciated in the r wave of V2 to V4 (>40 msec in atrioventricular pathways) [23].

**Figure 2:**
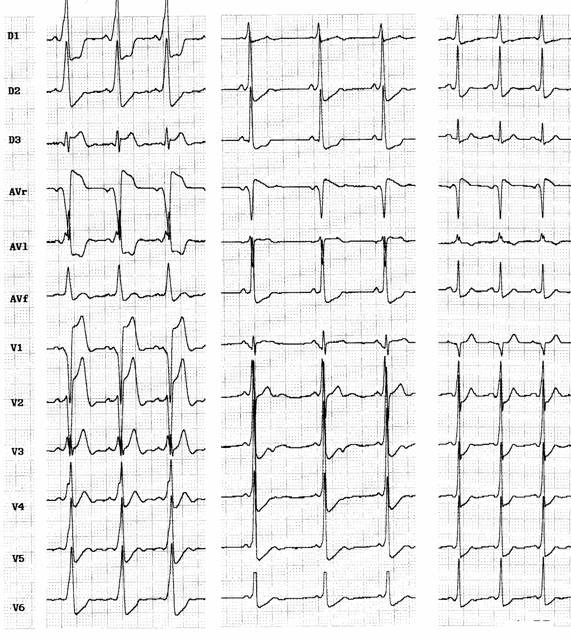
Three cases of fasciculoventricular pathways: PR intervals are 0,08 sec, 0,09 sec and 0,10 sec respectively. QRS is wider in the first ECG and <0.11 sec in the others 2 cases, and frontal plane axis are +30°, +75° and +45°. QRS transition occurs in V3, V2 and V2, respectively.

Electrocardiogram of fasciculoventricular pathway is characterized by normal frontal plane axis like an anteroseptal accessory pathway (0° to +75°) [27] with a subtle preexcitation and normal PR interval. It is also commom to see a short PR interval due to associated enhanced A-V nodal conduction. Atrial pacing do not change the degree of preexcitation. Junctional beats are preexcited and intravenous adenosine yields blocked P waves. Precordial transition (R/S >1) usually occurs at V2 (Figure 2).

### Electrophysiologic Characteristics

**Figure 3:**
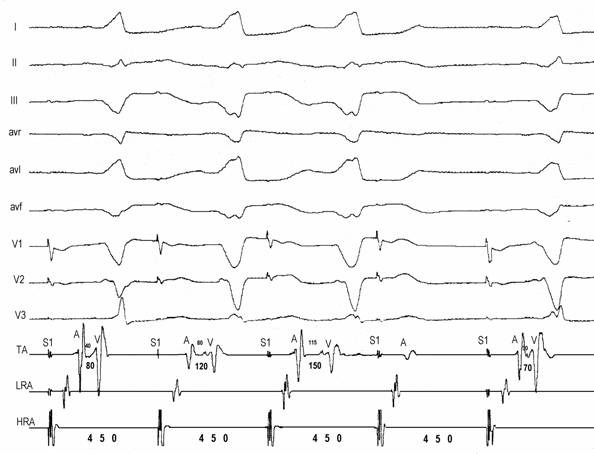
Atrioventricular pathway: high right atrial (HRA) pacing at 450 msec causes Wenckebach block on the accessory pathway. Progressive prolongation of AV interval (80-120-150-block) is due to prolongation of A-AP potential (40-80-115-block). Preexcitation degree increases from the first to the second QRS complex and remains constant in the third QRS despite further prolongation of the A-APP interval.

**Figure 4:**
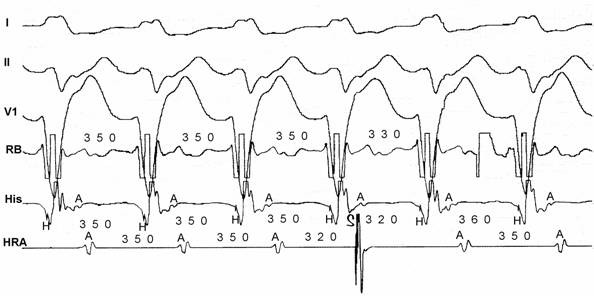
Antidromic tachycardia. Late (S) atrial extrastimuli delivered from the lateral high right atrium without disturbing AA timing at the His bundle recording advances QRS complex by 20 msec and His deflection by 30 msec, proving that the pathway is extra nodal and participates on the circuit.

The major findings are the slow conducting and decremental properties that can be assessed during right atrial pacing: AH interval lengthens, HV interval shortens and QRS widens until a steady value is achieved. Faster atrial stimulation does not increase preexcitation, but can prolong AV conduction time until block occurs (Figure 3). Decremental conduction is usually defined as rate dependent prolongation of conduction time in more than 30 msec through the accessory pathway (AP) as measured in the electrograms close to the AP insertion. Atrial extrastimulus testing produce likewise results increasing progressively AV interval and preexcitation up to a steady value. During atrial pacing, achievement of maximal preexcitation is associated with retrograde conduction over the right bundle, His bundle and A-V node and cessation of pacing is usually followed by antidromic tachycardia (Figure 4). The next step is to assess the role of the accessory pathway in the tachycardia circuit: active or bystander. It can be done delivering single late lateral right atrial extrastimuli during preexcited tachycardia, timed not to affect the His region and coronary sinus atrial electrogram at the ostium [11]. Advancement of QRS complex and atrial activation establishes the diagnosis of an extranodal accessory pathway (proximal atrial insertion) as well its involvement in the tachycardia circuit. If advancement of QRS activation occurs without changing of atrial activation, the presence of an extranodal AP is certain but its participation on the circuit is not. McClelland et al could successfully advance QRS activation with late right atrial extrastimuli in 22 of 23 patients with atriofascicular tachycardia [13]. Another less elegant maneuver proving AP participation in SVT is by producing catheter-induced RBBB. Assuming an antidromic tachycardia incorporating an atriofascicular (or atrioventricular) pathway retrograde conduction occurs via right bundle-His bundle and A-V node axis. RBBB lengthens the circuit path, tachycardia cycle length due to an increase in ventriculoatrial time. Preexcited A-V nodal reentry as well as antidromic tachycardia with retrograde conduction through another AP would not be affected. The proximal atrial insertion can also be localized with the recording of an accessory pathway potential, because it is usually recorded in the postero-lateral or antero-lateral aspect of tricuspid annulus, away from the A-V node. In the study of Grogin et al [28] clues as to the presence of a nodoventricular fiber were the inability of a premature atrial stimulus to advance the ventricle and the presence of dual A-V nodal pathways. Scheinman et al [29] were not agreeable with that statement and finding an "M" potential assumes diagnostic importance in this setting, because the "M" potential accurately localizes the anatomic site of the pathway.

Ventricular stimulation usually discloses ventriculoatrial conduction from the A-V node. The vast majority of atriofascicular and atrioventricular pathways have only anterograde conduction [30,31]. Adenosine injection during sinus rhythm can yield complete AV block or sometimes increase preexcitation [32]. Verapamil has a more proeminent effect over the A-V node, and can be of help in exposing preexcitation. During antidromic tachycardia adenosine causes prolongation of conduction over the pathway and eventual block, terminating tachycardia.

There are plenty of electrophysiologic and anatomic data supporting the concept that at least atriofascicular pathways are accessory AV nodes. We have seen a patient with an electrophysiologic profile suggestive of the presence of an atriofascicular pathway without conduction through the AV node. This patient had unexplained syncope with a baseline ECG showing LBBB-like pattern with normal PR interval (Figure 5a). Atrial pacing disclosed a decremental AP and 1:1 conduction up to 280 msec with an "M" potential in right posteroseptal region. There was no VA conduction. Adenosine yielded transient complete AV block. We decided to ablate the Mahaim-like pathway and as soon as radiofrequency current was delivered, the patient had developed complete AV block (Figure 5b). Ablation was immediately discontinued and patient resumed preexcitation. The transient escape rhythm after ablation had a normal HV interval but no conduction occurred over the AV node. This scenario is consistent with an ectopic accessory AV node without conduction from the "normal" AV node.

Associated conditions: Mahaim fibers (AF/ AV) very often occurs in the setting of Ebstein's disease (10 to 40%) and associated accessory pathways are a commom finding (up to 30%). Fasciculoventricular pathways also occurs very often in association with accessory pathways. I have reviewed the cases reported since 1981 [1,22,23,33,34] together with 3 cases from our own laboratory, and I found 6 of 15 patients with associated AP's (40%). Dual AV nodal pathways with AV nodal reentrant tachycardia is much higher of that expected by chance alone (Table 1).

## Treatment

### Mapping and Catheter Ablation

**Figure 6:**
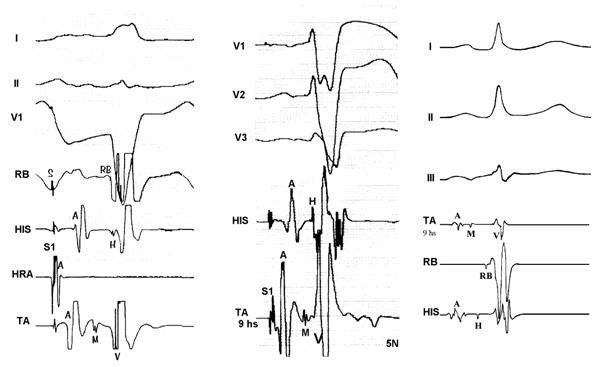
Three examples of "M" potentials (TA- tricuspid annulus electrograms): from left to right: first two cases with His-like potentials and the third with narrow and low amplitude potential. Ablation was successful in each of those sites.

**Figure 7:**
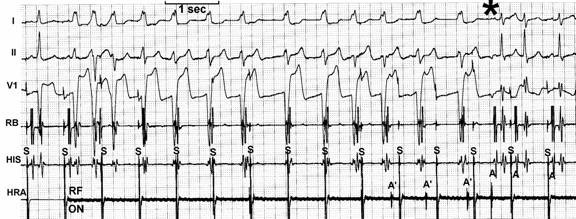
Radiofrequency catheter ablation during atrial pacing: MAT starts immediately after current delivery (RF-ON) and lasted for 8 seconds. Preexcitation is no longer present when MAT terminates.

**Figure 8:**
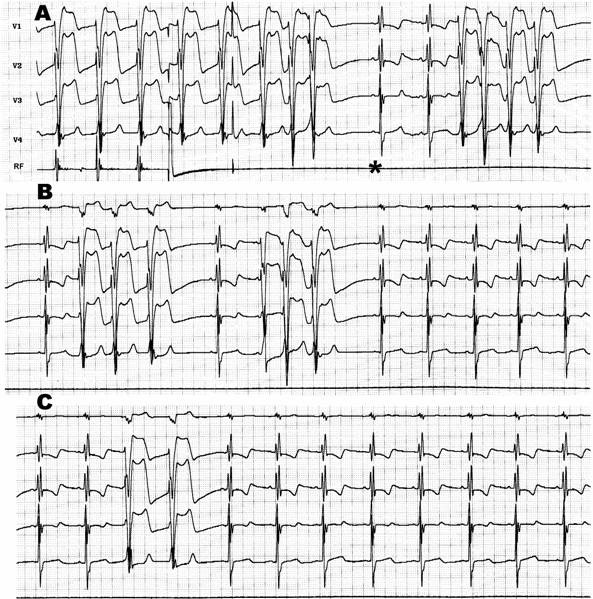
Radiofrequency ablation of recurrent atriofascicular pathway conduction: during ablation salvos of automatic and irregular rhythms with the same QRS morphology as the preexcited one. The salvos persisted even after abolition of preexcitation (*) and fade out until complete disappearance.

Some particular features are unique to Mahaim fibers: Mapping of the atrial insertion by ventricular stimulation is usually not possible because those decremental pathways do not conduct retrogradely. Atrioventricular connections can be located by mapping the site of earliest ventricular activation on the annulus, as with other anterogradely conducting accessory AV pathways. On the contrary, atriofascicular pathways or even the long atrioventricular pathways with distal (nonannular) insertion cannot be mapped in this way. To worse matters these decremental pathways are unusually sensitive to mechanical trauma. Inadvertent knocking of the ablation catheter against the annulus can result in transient abolition of conduction through the pathway from minutes to hours [34,35]. The following strategies have been used to overcome those problems:

1. Searching for the "M" (Mahaim) potential (Figure 6) along the tricuspid annulus is the most commonly used technique. The ablation catheter should be carefully moved along the annulus avoiding bumps on the tissue. We routinely use a long sheath like DAIG^®^ SR2 or SR3, which improves stability. The potential may be as large as the His bundle potential or small, narrow with low amplitude. Catheter ablation at a site with "M" potential is likely to be successful (Table 1). We [20] and other authors [19,29] have recorded automatic rhythms ("Mahaim" automatic tachycardia-MAT) brought about during radiofrequency current delivery. It is probably due to heat-related automaticity of nodal-like tissue in a similar fashion to junctional rhythm that arises during slow A-V nodal pathway ablation. It seems to represent a hallmark for successful ablation particularly of atriofascicular pathways. MAT in most cases is short-lived (Figure 7), but ocasionally it lasts longer. We have had an early out of hospital recurrence when it was not our policy to completely eliminate such rhythm. In a second procedure we decided to ablated until complete elimination of automatic activity (Figure 8). We have seen MAT during radiofrequency catheter ablation of atriofascicular pathway but not with atrioventricular pathways.
2. Activation mapping of the earliest local ventricular potential is feasible in short atrioventricular pathways like in fast conducting AV accessory pathways. Atrioventricular decremental pathways with a long course often shows extensive arborization over a wide area of ventricular muscle [23]. Targeting distal branches is a time consuming task. It is possible to ablate some of them, as assessed by changes in preexcitation pattern, but a complete elimination is very unlikely. Some patients with atriofascicular pathway who underwent ablation at the distal insertion had developed a proarrhythmic [13,35] response with facilitation of antidromic tachycardia occurrence due to slow conduction induced by radiofrequency ablation.
3. Shortest stimulus-QRS interval as assessed by atrial stimulation at a constant pacing rate along the atrial aspect of the annulus was the gold standard mapping method before mapping of "M" potential had been reported. Stimulation sites remote from the atrial insertion of the accessory pathway result in long stimulus-QRS interval due to the amount of interposed atrial tissue. We do not use this method because it is time consuming and very inaccurate because it is difficult to stimulate from many sites at the same distance from the annulus and stimulating atrial tissue requires good contact with the tip, which is not always possible.
4. Extrastimulus mapping during antidromic tachycardia. Finding an atrial site where the longest coupled premature extrastimulus causes resetting, or assessing the amount of advancement of the QRS following application of a fixed atrial extraestimulus coupling interval. Similar to the previous technique it looks for a site in the atrial annulus with the least interposing tissue separating it from the accessory pathway proximal insertion. Likewise shortest stimulus-QRS technique is an inaccurate and tedious method.
5. Some authors [36,37] reported the judicious use of mechanical trauma in a controlled way and transient AP conduction block to find the AP insertion. The rationale of this method is based on the observation that transient conduction block following bumps of the catheter on the atrial aspect of the annulus is a frequent phenomena in decremental anterograde pathways. Gentle pressure of the tip of a steerable mapping catheter during antidromic tachycardia or atrial pacing would lead to transient block of AP conduction locating the site for ablation. The fact that those AP are more prone to mechanical trauma suggests that they may be thinner and have a more superficial location than others. The main pitfalls are: when conduction block occurs the catheter is on the move and may be away from the AP site, and its relocation will be dependent on resuming of AP conduction; mechanical block can last hours and a second procedure would be needed. I do not favor the use of this method and I'd rather avoid mechanical trauma.
6. Electroanatomic mapping (noncontact mapping) [34,38] can be helpful, while not widely available, in those cases where accessory pathway potential cannot be found and when mechanical trauma precludes adequate mapping. This technique allows the operator to "tag" the exact location of the tip of the catheter and in case of transient conduction block the catheter can be manipulated back to the tagged location for ablation

Radiofrequency current should be applied during atrial pacing to enhance preexcitation and making it easier to assess conduction block at the AP. Stability is improved during atrial pacing as compared with ablation during antidromic tachycardia when catheter is likely to move with tachycardia termination. MAT is a common and expected event and can also cause catheter displacement. Before "M" potential mapping technique became the gold standard mapping technique some authors favored targeting the ventricular insertion to avoid MAT and maintain a better catheter stability [39].

Catheter ablation have been very successful particularly when ablating at a site with "M" potential or assessing earliest delta-V interval in atrioventricular decremental pathways (Table 1).

## Figures and Tables

**Figure 5a:**
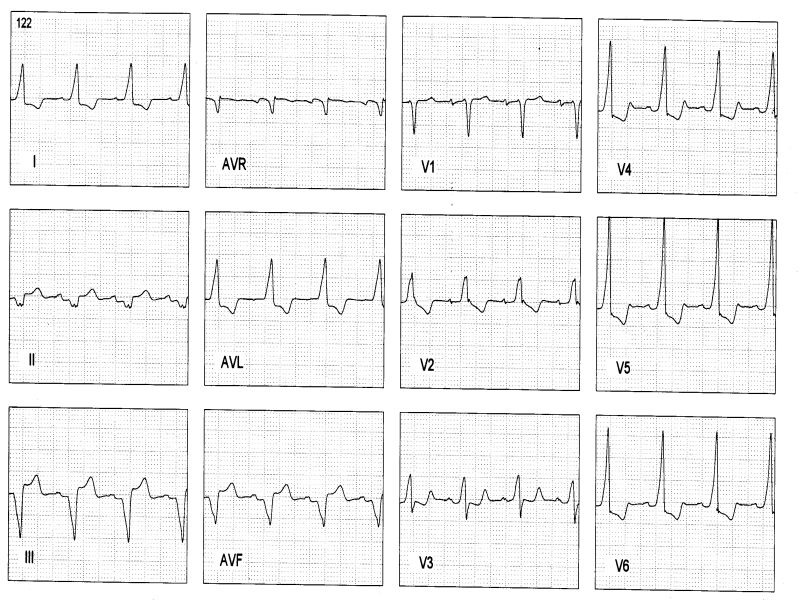
Baseline ECG disclosing a normal PR interval (0,16 sec) and a preexcited QRS complex consistent with a right posteroseptal decremental AP

**Figure 5b:**
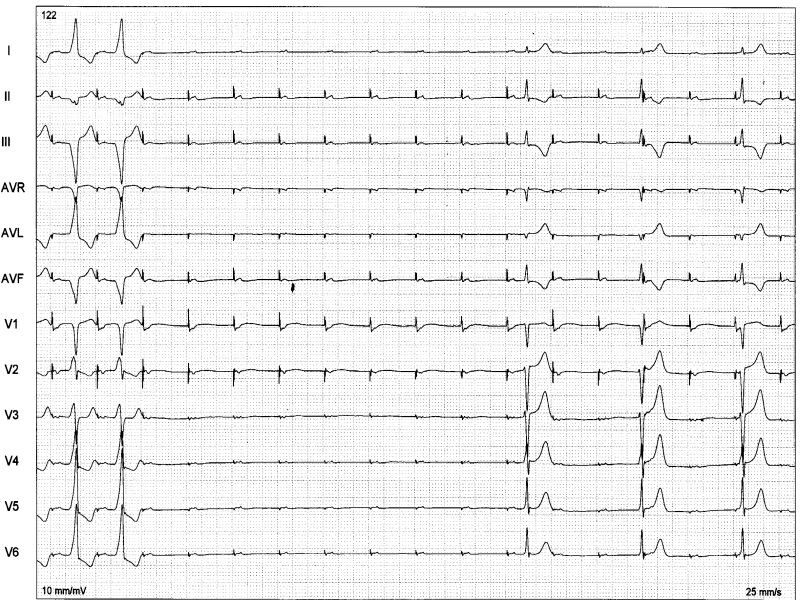
During atrial pacing a brief pulse (1 sec) of radiofrequency current delivered at a site with "M" potential yield a transient complete AV block followed by an escape rhythm without preexcitation (30-35 b/min). Preexcitation resumed in 14 seconds

**Table 1:**
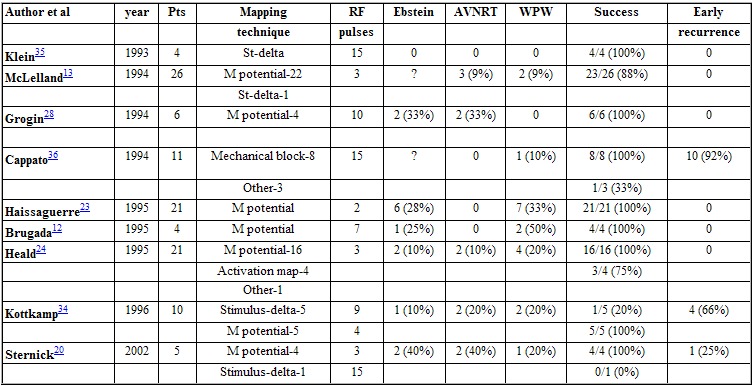
Data from those series suggest that authors using the "M" potential as the mapping technique for ablation are more successful with less RF pulses and lower recurrence rate.
